# Recurrent Mitral Valve Endocarditis Caused by *Streptococcus pneumoniae* in a Splenectomized Host

**DOI:** 10.1155/2013/929615

**Published:** 2013-12-28

**Authors:** Shikha Shrestha, JayaKrishna Chintanaboina, Samir Pancholy

**Affiliations:** ^1^Wright Center for Graduate Medical Education, 501 Madison Avenue, Scranton, PA 18510, USA; ^2^Penn State Hershey Medical Center, 500 University Dr, Hershey, PA 17033, USA

## Abstract

A 72-year-old male with a remote history of splenectomy and two previous episodes of pneumococcal endocarditis of mitral valve presented with high-grade fever and confusion for 3 days. Nine months priorly, patient underwent mitral valve repair when he had the first episode of pneumococcal mitral valve endocarditis. He received pneumococcal vaccination two years ago. On examination during this admission, he was found to be febrile (104.3 F) and confused and had a grade 2/6 systolic murmur at the apex without any radiation. Laboratory data was significant for a white blood cell count of 22,000/mm^3^ (normal: 4000–11000/mm^3^). Blood cultures (4/4 bottles) grew penicillin-sensitive *Streptococcus pneumoniae*. Transesophageal echocardiogram revealed small vegetation on the posterior mitral leaflet without any evidence of abscess and severe mitral regurgitation. Patient clinically responded to intravenous ceftriaxone. However, due to recurrent pneumococcal mitral valve endocarditis and severe mitral regurgitation, the patient underwent mitral valve replacement. Patient had an uneventful recovery and was discharged home. Pneumococcal endocarditis itself is being uncommon in this current, penicillin, era; our case highlights the recurrent nature of pneumococcal endocarditis in a splenectomized host and the importance of pursuing aggressive treatment options in this clinical scenario.

## 1. Introduction

Pneumococcal endocarditis (PE) has become relatively uncommon since the advent of penicillin. However, it is a clinically relevant disease because of its significant mortality rate which ranges between 25 and 50% [1]. Although pneumococcal bacteremia is quite common in immunosuppressed/splenectomized patients, the incidence of pneumococcal endocarditis itself is very rare in this group of patients [2]. We report a case of recurrent pneumococcal mitral valve endocarditis in a splenectomized patient.

## 2. Case Report

**Figure 1:**
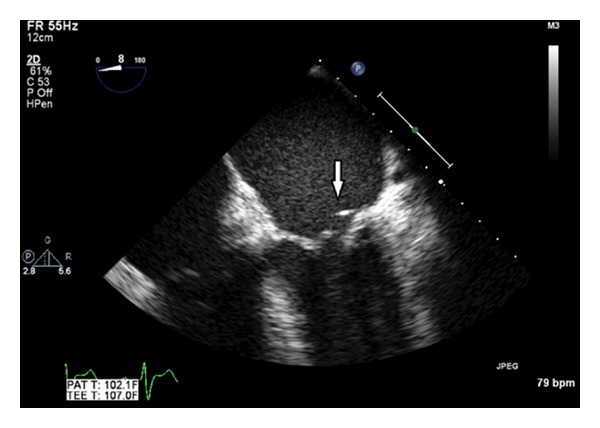
Transesophageal echocardiogram. A small vegetation (white arrow) on the mitral leaflet at the site of mitral valve repair.

A 72-year-old male with a remote history of splenectomy secondary to Hodgkin's lymphoma, paroxysmal atrial fibrillation (on anticoagulation), coronary artery disease status after coronary artery bypass graft (X1 vessel), and hypertension presented with high-grade fever and confusion for 3 days. He had similar complaints 9 months prior, at which time he was diagnosed with pneumococcal meningitis and pneumococcal mitral valve endocarditis (often referred to as Austrian syndrome). During that time, a transesophageal echocardiogram (TEE) showed mitral valve vegetation and posterior annular abscess. Blood cultures grew penicillin-sensitive *Streptococcus pneumoniae*. He underwent mitral valve repair, which consisted of P2 resection with the vegetation and repair of the posterior annular abscess at the hospital of the University of Pennsylvania (HUP). Five months later, he was again diagnosed with penicillin-sensitive pneumococcal mitral valve endocarditis; however, he responded promptly to medical therapy with ceftriaxone given intravenously over a 6-week period. He had received a pneumococcal vaccination 2 years prior to this presentation. On physical examination during this admission, he was alert but disoriented to time, place and person. The temperature was 104.3°F, blood pressure 120/74 mm Hg, heart rate 87/minute, and respiratory rate 22/minute. Cardiac auscultation revealed a soft S1 and grade 2/6 systolic murmur at the mitral area without any radiation. There were no meningeal signs, focal neurological deficits, or peripheral stigmata of infective endocarditis. Laboratory data is shown in Table 1. Chest X-ray and urinalysis were normal. Electrocardiogram showed an old left bundle branch block. Computed tomography of head was normal. The patient was initially started on intravenous ampicillin, ceftriaxone, and vancomycin while waiting for blood cultures. Lumbar puncture was not performed on admission due to the risk of bleeding as the patient was on coumadin (INR-2.3). Blood cultures (4/4 cultures) grew penicillin-sensitive *Streptococcus pneumoniae*. Ampicillin and vancomycin were stopped on day 3. TEE revealed severe mitral regurgitation and a small vegetation on the posterior mitral leaflet without any evidence of abscess (Figure 1). Patient clinically responded to intravenous ceftriaxone. However, due to recurrent pneumococcal mitral valve endocarditis and severe mitral regurgitation, the patient underwent mitral valve replacement. There was a vegetation on the pledgeted suture that was placed during mitral valve repair, and the whole suture line along P2 was infected. This infected area was widely debrided. A #29 St. Jude mechanical valve was placed. The patient had an uneventful postoperative recovery and was discharged home on anticoagulation for atrial fibrillation and mechanical heart valve. He finished a 6-week course of intravenous ceftriaxone therapy. During the follow-up visit at the clinic 6 weeks later, he was found to be asymptomatic and stable.

## 3. Discussion

*Streptococcus pneumoniae* is a normal member of the respiratory flora and its invasion often leads to pneumonia. Invasive pneumococcal disease is still a significant cause of mortality and morbidity in this 21st century [3]. *Streptococcus pneumonia* is recognized to be the most common cause of community-acquired pneumonia and as such, pneumococcal bacteremia is usually the sequelae of lung infection. Less commonly, it may follow sinusitis and otitis media and at times, there is no recognized focus of infection [1–4].

PE has been a rare clinical entity since the advent of penicillin in the 1940s. However, the morbidity and mortality associated with this clinical entity is high [4]. As most of the literature is derived from case reports and small case series, the incidence of PE is not exactly known but few clinical studies have estimated the prevalence to be less than 3% [1, 5, 6]. Recurrence of PE is even more rare. Very few cases of recurrent PE are reported in the medical literature. To our knowledge, the last reported case of recurrent PE was by Lindberg and Fangel in 1999 [7]. Risk factors of PE include extremes of age, alcoholism, malnutrition, immunosuppression, and prior valvulopathy. Local factors that cause impaired clearance mechanisms in the lung tissue, such as chronic tobacco use, chronic pulmonary disease, and recent lung infection, are other identified risk factors [1, 8, 9]. Alcoholism is considered to be one of the strongest risk factors for PE [9], although our patient denied any significant alcohol consumption; immunosuppression secondary to splenectomy and old age were considered to be the predisposing factors.

The most common presenting symptom of PE is fever. Diagnosis is often delayed due to concomitant infectious processes [10, 11]. PE tends to be very aggressive and is associated with substantial mortality rates. Tanawuttiwat et al. reported a case of pneumococcal mitral valve endocarditis that had a fulminant course with extremely rapid valve destruction [2]. Given its acute presentation, the peripheral stigmata of infective endocarditis are rarely seen [2, 5, 12]. It can affect either native or prosthetic valves and has a predilection for the aortic valve. The mitral valve is reported to be the second most commonly affected valve in PE [1, 4, 10]. At times, multiple valves can be affected simultaneously [1]. Our patient had recurrent acute infection of the mitral valve and did not have any signs of the peripheral stigmata of infective endocarditis on presentation during any of the three episodes.

The most frequent complications of PE in adults are congestive heart failure, large arterial emboli, and focal abscesses [4]. Given its acute presentation and rapid course, patients should be closely monitored for any complications. Echocardiogram is the cornerstone in the diagnosis of infective endocarditis [1, 4, 10]. Transthoracic echocardiograms (TTE) have been shown to detect vegetations in about 90% of cases of pneumococcal endocarditis due to its propensity to form large vegetations [1, 4]. On the other hand, the rates of the detection of complications with TTE for valvular perforation and abscess are 20% and 13%, respectively [1, 4]. TEE is far more sensitive than TTE for the detection of the extent of valve damage and, hence, TEE should be performed in all PE patients as its findings may alter the management approach [1, 4, 10].

Treatment of PE includes a prolonged course of intravenous antibiotics and, if needed, surgery [13]. Penicillin was the drug of choice for treatment of PE in the past. However, due to the increase in the incidence of penicillin-resistant strains and frequent association with meningitis [10, 14, 15], a 3rd generation cephalosporin and vancomycin are often started on admission depending on the clinical scenario. An association of PE with pneumococcal meningitis (and at times, pneumococcal pneumonia) is often referred to as Austrian syndrome. Few cases of Austrian syndrome are reported in the medical literature [16, 17]. Martínez et al. reported a concerning increase in the resistance of pneumococci to penicillin, but the study did not show any effect of penicillin-resistant strains on clinical characteristics, complications, and mortality. However, left-sided heart failure was associated with increased mortality in this study [10]. Surgical evaluation must be pursued in any PE patient with suspicion for complications like abscess, large vegetation, and valve perforation. The combined medical and surgical approach has improved outcomes, as compared to medical treatment alone in this group of patients [10, 13]. Pneumococcal vaccine should be administered to all at-risk patients to prevent complications and recurrence of PE [18, 19]. Our patient was prone to pneumococcal bacteremia and endocarditis even though pneumococcal vaccine was administered.

## 4. Conclusion

Our case report emphasizes that high index of suspicion for PE is required in any immunosuppressed patient who presents with high-grade fever and pneumococcal bacteremia. Early recognition with echocardiography, prompt initiation of appropriate antibiotic therapy, and surgical evaluation would help decrease mortality in this group of patients. A few studies have shown that mitral valve repair may have better long-term results in mitral valve endocarditis [20, 21]; however, our case emphasizes that mitral valve replacement may be a better option over mitral valve repair during the initial episode of pneumococcal mitral valve endocarditis in an immunosuppressed patient due to its aggressive course and propensity to recur. However, further investigation is required to support this recommendation. Although the efficacy of pneumococcal vaccine was debatable in our patient, it should be promptly administered to all susceptible patients as per standard immunization guidelines.

## Figures and Tables

**Table 1:** 

Laboratory test	Result	Laboratory reference
White blood cell	22,000	4000–11000 cells/cc
Neutrophils		
Bands		
Lymphocytes		
Hemoglobin	10.8	
Hematocrit	32.7	
Serum sodium	135	135–145 meq/dL
Serum potassium	4.4	3.5–4.5 meq/dL
Serum bicarbonate	23	22–24 meq/dL
Serum chloride	103	100–110 meq/dL
Serum creatinine	1.48	0.6–1.2 mg/dL
Blood urea nitrogen	41	8–24 mg/dL
Serum calcium	9.4	8.7–10.2 mg/dL
Blood glucose	124	140–180 mg/dL
Prothrombin time	24.8	
International normalized ratio (INR)	2.3	
